# Controllable Structure and Fluorescence Enhancement of ACQ Dye Nanoparticles Based on the FNP Process

**DOI:** 10.3390/polym17152152

**Published:** 2025-08-06

**Authors:** Yue Wu, Yutao Zhang, Zhiqian Guo, Yisheng Xu

**Affiliations:** 1 Center of Photosensitive Chemicals Engineering, State Key Laboratory of Green Chemical Engineering and Industrial Catalysis, State Key Laboratory of Chemical Engineering, School of Chemical Engineering, East China University of Science and Technology, Shanghai 200237, China; 2 Key Laboratory for Advanced Materials and Joint International Research Laboratory of Precision Chemistry and Molecular Engineering, Feringa Nobel Prize Scientist Joint Research Center, Institute of Fine Chemicals, School of Chemistry and Molecular Engineering, East China University of Science and Technology, Shanghai 200237, China

**Keywords:** fluorescence nanoparticles, ACQ effect, flash nanoprecipitation, fluorescence enhancement

## Abstract

Fluorescent dyes, such as cyanine dyes, are widely used in fluorescence-imaging-guided tumor therapy due to their high absorbance and fluorescence quantum yield. However, challenges persist in optimizing the performance of fluorescent nanoparticles, particularly due to the aggregation-caused quenching (ACQ) effect of cyanine dyes. Here, a novel counterion construction strategy is introduced using cyanine dye as a model ACQ dye. Through dynamic-controlled flash nanoprecipitation, fluorescent nanoparticles (CyINPs) with tunable structures are developed, investigating the effects of various factors, including counterions, block copolymers, and dye concentrations, on CyINPs’ stability and fluorescence enhancement. The optimized CyINPs with good water solubility show a 21-fold increase in fluorescence intensity and a 3.5-fold increase in encapsulation efficiency compared to CyINPs prepared by a thermodynamic-driven method. Under the efforts of polymers and counterions, dyes are separated, which reduces the impact of the ACQ effect and results in stronger fluorescence intensity, providing insights into improving nanoparticle biocompatibility and energy utilization efficiency.

## 1. Introduction

Cancer, recognized as one of the most lethal diseases worldwide, leads to a mortality rate surpassed only by heart disease and infectious diseases [1,2,3,4]. With the rapid development of modern science and technology, nanoparticles have become a research hotspot in the field of biomedicine. These advancements facilitate the precise spatial and temporal distribution of imaging and therapeutic agents at the disease site, thus laying the theoretical and experimental groundwork for diagnostic and therapeutic integration [5,6,7,8,9,10].

Cyanine dye, as a fluorescent dye with high absorbance and high fluorescence quantum yield, is commonly used in fluorescence-imaging-guided tumor therapy [11,12,13,14,15,16,17]. This approach typically involves the combination of anti-tumor drugs with photodynamic or photothermal effects with fluorescent dyes, which are subsequently loaded into nanoparticles [18,19,20,21]. However, the application of these fluorescent nanoparticles still faces challenges in efficient imaging-guided tumor therapy. One key issue is the aggregation-caused quenching (ACQ) effect of cyanine dyes, which makes the fluorescence signal intensity highly sensitive to the dispersed state of the dyes [22,23,24,25,26]. This sensitivity limits both the diagnostic signal intensity and the therapeutic efficacy, as the self-assembly process of cyanine dyes may hinder the interaction of functional molecules, affecting both stability and effectiveness. Additionally, conventional nanoparticle preparation methods usually require long processing times and uncontrollable particle structure [27,28]. Therefore, there is an urgent need to develop a controllable structure preparation strategy to achieve fluorescence enhancement of ACQ cyanine dye nanoparticles while simultaneously improving energy utilization efficiency.

**Scheme 1:**
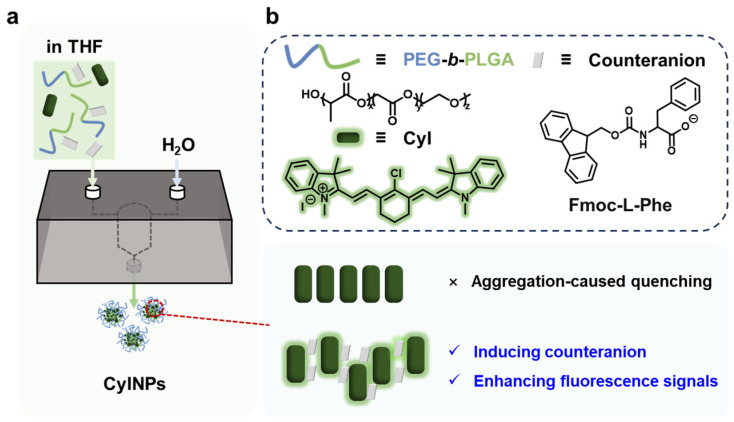
(**a**,**b**) Preparation of CyINPs and the fluorescence enhancement strategy.

Herein, a controllable-structural counterion construction strategy is developed utilizing CyI dye as a model ACQ cyanine dye, which is prepared by dynamic-controlled flash nanoprecipitation (FNP). CyI fluorescent nanoparticles (CyINPs) with controllable structures are formed by introducing counterion molecules and block copolymers (BCPs), thereby realizing dispersed structure of CyI dyes. The effects of counterion molecules, BCPs, and dye concentrations, respectively, on the stability of CyINPs, as well as the enhancement of fluorescence intensity, are investigated. Structural adjustments of CyINPs are achieved by optimizing FNP parameters, which both improve CyINP encapsulation efficiency and enhance the fluorescence intensity of CyINPs. Furthermore, the influence of the structure of CyINPs on fluorescence enhancement is explored, providing insights into the improvement of the biocompatibility of fluorescent nanoparticles and the efficient utilization of energy (Scheme 1).

## 2. Materials and Methods

### 2.1. Materials

The CyI dye was synthesized as previously reported [29]. The amphiphilic block copolymer PEG(5k)-*b*-PLGA(10k) was purchased from Yarebio (Shanghai, China). Triptycene, sodium tetraphenylboron, sodium tetrakis [3,5-bis(trifluoromethyl)phenyl]borate (NaBARF), L-phenyalanine, Fmoc-L-phenyalanine (Fmoc-L-Phe), tetrahydrofuran (THF), and dimethyl sulfoxide (DMSO) were purchased from Shanghai Tansole Chemical Co., Ltd. (Shanghai, China). 3-(4,5-dimethylthiazol-2-yl)-2,5-diphenyltetrazolium bromide (MTT), Dulbecco’s modified Eagle’s medium (DMEM), and phosphate-buffered saline (PBS) were purchased from Keygentec (Nanjing, China). Fetal bovine serum (FBS) and penicillin–streptomycin liquid were purchased from Gibco (Billings, MT, USA). Ultrapure water was used in all experiments and was prepared using a Milli-Q water purification system.

### 2.2. Characterization

Dynamic light scattering (DLS) was performed at 25 °C using a Malvern Zetasizer Pro Blue ZSU3200 (Malvern Panalytical, Malvern, UK) with a fixed angle of 90°. The polydispersity index (PDI) is a dimensionless measure of the broadness of the size distribution, calculated from parameters’ fit to the correlation data through cumulant analysis, which is defined in the ISO standard document 13321:1996 E and ISO 22412:2008 [30,31]. Absorption spectra were investigated with an Agilent Technologies Carry 60 UV–Vis spectrometer (Agilent, Santa Clara, CA, USA), while fluorescence spectra were taken on a Cary Eclipse fluorescence spectrometer (Agilent, Santa Clara, CA, USA). Transmission electron microscopy (TEM) was conducted on a JEOL JEM-2100 TEM instrument (JEOL, Tokyo, Japan) operating at 300 kV, and scanning electron microscopy (SEM) images were acquired using a Nova NanoSEM 450 microscope (Thermo Fisher Scientific, Waltham, MA, USA). X-ray diffraction (XRD) data were recorded with a Bruker D8 XRD diffractometer (Bruker, Billerica, MA, USA) using Cu Kα radiation (40 kV, 40 mA) over a 2θ range of 5–80° with 0.02° increment. Thermogravimetric analysis (TGA) was performed on an STA 449/F3 thermal analyzer (NETZSCH, Selb, Germany) at a heating rate of 5 °C/min in air. Confocal laser scanning microscopy (CLSM) images were obtained using a Nikon AIR laser scanning microscope (Nikon, Tokyo, Japan).

### 2.3. Synthesis of CyINPs by FNP

CyINPs-FNP were prepared by a confined impinging jet mixer (CIJ-D) through the flash nanoprecipitation (FNP) method. A solution of CyI (0.5–2.0 mg·mL^−1^) in tetrahydrofuran (THF) was mixed with PEG-*b*-PLGA block copolymers (0.5–5.0 mg·mL^−1^) in THF. The two streams were introduced at controlled flow rates ranging from 2 to 50 mL·min^−1^ using Harvard Apparatus PHD 2000 syringe pumps (Harvard Apparatusto, Holliston, MA, USA) to initiate nanoprecipitation. The resulting suspensions were dialyzed against deionized water using regenerated cellulose dialysis membranes with an MW cutoff of 3.5 kDa for 24 h, followed by dispersion in ultrapure water.

### 2.4. Synthesis of CyINPs by Thermodynamic-Driven Method

For comparison, CyINPs-TM were prepared by a thermodynamically driven method. Briefly, 0.5 mg of CyI and 10 mg of PEG-*b*-PLGA were dissolved in 1 mL of THF. Then, the mixture was added to 9 mL of water under vigorous stirring. The resulting CyINPs-TM suspension was also dialyzed in pure water for 24 h.

### 2.5. Encapsulation Efficiency of CyINPs

The encapsulated CyI of CyINPs was characterized by UV–Vis spectroscopy. CyINPs were first lyophilized and then dissolved in DMSO. The absorption spectra of this solution were measured and compared with the calibration curve of CyI (Figure S1), from which the amount of CyI was calculated.

(1) $$\mathrm{encapsulation}\;\mathrm{efficiency}\;(\%)=\frac{\mathrm{the}\;\mathrm{amount}\;\mathrm{of}\;\mathrm{loaded}\;\mathrm{CyI}}{\mathrm{the}\;\mathrm{amount}\;\mathrm{of}\;\mathrm{CyI}\;\mathrm{added}}\;\times \;100\;\%$$

### 2.6. Cell Culture

HUVECs (Human Umbilical Vein Endothelial Cells) and HeLa cells used to investigate the in vitro performance of CyINPs were obtained from the Institute of Biochemistry and Cell Biology, Shanghai Institutes for Biological Sciences (SIBS), Chinese Academy of Sciences (CAS). All cells were cultured in DMEM supplemented with 10% FBS and 1% antibiotic/antimycotic solution at 37 °C under 5% CO_2_.

### 2.7. Cytotoxicity of CyINPs

In vitro cytotoxicity was measured through methyl thiazolyl tetrazolium (MTT) assay as follows.

#### 2.7.1. Dark Cytotoxicity of CyINPs

HUVECs and HeLa cells were incubated in 96-well plates (1 × 10^4^ cells/100 μL per well) containing DMEM for 12 h. The medium was next replaced by fresh DMEM containing different concentrations (0, 2, 4, 6, 8, 10, and 16 μM) of CyINPs-FNP and CyINPs-TM, respectively, where 0 μM refers to PBS acting as the control group. After 12 h incubation, MTT solution (50 μL) was added, followed by a further 4 h incubation. Then, 150 μL of DMSO was added to each well. Finally, the optical density OD 570 value (Abs) of each well with background subtraction at 490 nm was measured using a Tecan Infinite M200 monochromator-based multifunction microplate reader (Tecan, Männedorf Switzerland).

(2) $$\mathrm{Cell}\;\mathrm{Viability}\;(\%)=\frac{\mathrm{mean}\;\mathrm{Abs}\;\mathrm{value}\;\mathrm{of}\;\mathrm{treatment}\;\mathrm{group}}{\mathrm{mean}\;\mathrm{Abs}\;\mathrm{value}\;\mathrm{of}\;\mathrm{control}\;\mathrm{group}}\;\times \;100\;\%$$

#### 2.7.2. Cytotoxicity of Photothermal Effect Caused by CyINPs

HeLa cells were cultivated in 96-well plates at a density of 1 × 10^4^/100 μL containing DMEM for 12 h. The cells were incubated with CyINPs (0, 1, 5, 10, 25, and 50 μM) for 30 min, then exposed to 808 nm laser irradiation (0.5 W∙cm^−2^, 5 min). MTT assay was conducted as described above in Section 2.7.1.

### 2.8. Statistical Analysis

All data were presented as mean ± standard deviation (SD) with n ≥ 3. Statistical significance was evaluated using one-way ANOVA through Origin 2018 software. Significance levels were set as: * *p* < 0.05; ** *p* < 0.01; *** *p* < 0.001.

## 3. Results and Discussion

CyI cyanine dye, synthesized as previously reported, exhibits strong absorbance and fluorescence quantum yields in organic solvents [29]. However, a strong aggregate-caused quenching (ACQ) effect limits the application of CyI, as CyI dyes are extremely sensitive to aggregate states. As shown in Figure S2, free CyI has good solubility and shows a dispersed state in DMSO, producing a bright green solution. In contrast, free CyI has lower solubility in THF and presents a partially aggregated state, thus causing a color change in the solution. Nevertheless, since free CyI is insoluble in H_2_O, free CyI precipitates heavily in H_2_O, leaving a solution in the aggregated state that is green with a hint of blue. Additionally, the absorbance and fluorescence intensity of CyI dyes decrease with the addition of H_2_O, which is caused by poor solubility of CyI and the increased aggregation state of CyI, thus exhibiting a more obvious ACQ effect. Therefore, in order to obtain the CyI in the dispersed state even in aqueous solution, counterion molecules and polymers are introduced to separate the CyI dyes preventing aggregation, leading to the enhancement of CyI fluorescence in H_2_O.

CyI nanoparticles (CyINPs) are prepared by CyI, counterion molecular, and block copolymers (BCPs) via the flash nanoprecipitation (FNP) method. Through the FNP method, different counterion molecules and process parameters could be manipulated [32,33,34,35,36,37], which enhances the CyINPs’ fluorescence intensity, avoiding the ACQ effect.

### 3.1. Selection of Counterion Molecules

Counterion molecules, including sodium tetraphenylborate (NaTPB), sodium tetrakis(3,5-bis(trifluoromethyl)phenyl)borate (NaBARF), and trichothecene (Trip) with different charges as well as steric hindrance, are used to prepare different CyINPs. As shown in Figure S3, the particle size of CyINPs with Trip and without counterions before dialysis exceeds 200 nm, and the PDI is higher than 1.5, and it is generally accepted that nanoparticles with a dispersion below 0.3 are monodisperse. Meanwhile, sedimentation of CyI dyes can be observed for CyINPs with Trip and without counterions after dialysis (Figure S4a), attributed to the hydrophobicity of CyI and the lack of electrostatic interaction with electrically neutral Trip. Furthermore, the particle sizes of BARF-CyINPs and TPB-CyINPs are around 100 nm, with particles stabilized for more than one week. Notably, BARF-CyINPs demonstrate higher encapsulation efficiency but poorer particle dispersion than TPB-CyINPs (Figures S4 and S5). This may be because the potential of BARF is more negative compared to TPB, which binds more CyI dyes, and thus CyINPs are more aggregated in preparation. The encapsulation efficiency is calculated through a calibration curve of CyI (Figure S1). As shown in Figure S6, the absorption and fluorescence spectra demonstrate a lower aggregation state of CyI in CyINPs than free CyI in THF. Since TPB-CyINPs have a stronger fluorescence intensity, a series of CyINPs is prepared under different CyI to TPB molar ratios, showing that as the molar ratio increases, fluorescence intensity is inversely related to encapsulation efficiency (Figures S7–S9). This is due to the fact that high CyI loading decreased dye–dye proximity, promoting the ACQ effect.

Therefore, counterions with weaker steric hindrance, such as L-phenylalanine (L-Phe), are explored to improve fluorescence intensity while maintaining encapsulation efficiency. As shown in Figures S10–S12, increasing the ratio of L-Phe initially leads to a gradual decrease in the particle size of CyINPs, followed by an increase. Additionally, the trend is accompanied by an increase in encapsulation efficiency and a decrease in fluorescence intensity. For BARF and TPB, L-Phe reduces the steric hindrance and weakens the electrostatic interaction with CyI, making the stacking state of CyI more dispersed, which consequently leads to stronger fluorescence signal intensity at this time than that of BARF-CyINPs and TPB-CyINPs.

### 3.2. Effect of Fmoc-L-Phe Anionic Ligand Ratio on Fluorescence Enhancement

Since L-Phe interacts with CyI by electrostatic and hydrophobic interactions to form the hydrophobic core, CyI is stabilized in CyINPs. On this basis, the Fmoc group is introduced into L-Phe in an attempt to make CyI retain the more dispersed state to achieve fluorescence enhancement while introducing part of the aggregated state of CyI to make CyINPs have certain photothermal properties.

**Figure 1:**
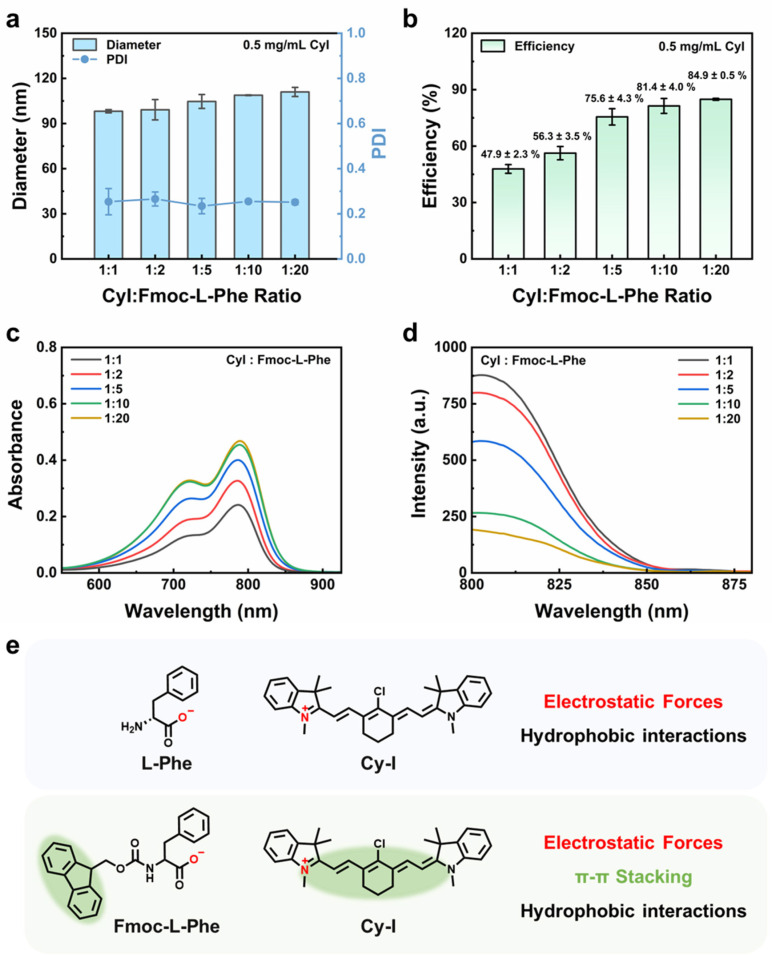
(**a**) Particle size, PDI, and (**b**) encapsulation efficiency of CyINPs with different CyI–Fmoc-L-Phe mole ratios. (**c**) Absorption spectra and (**d**) emission spectra of CyINPs with different CyI–Fmoc-L-Phe mole ratios ($\lambda_{ex}$ = 785 nm). (**e**) Interaction between CyI and L-Phe and Fmoc-L-Phe.

As shown in Figure 1a,b and Figure S13, the particle size and encapsulation efficiency of CyINPs increase with the increase of Fmoc-L-Phe concentration. This is due to the π-π stacking effect between the Fmoc group and CyI dye, which binds more CyI dye when the concentration of Fmoc-L-Phe increases. Thus, the growth of the hydrophobic core of the CyINPs becomes faster, and the polymer cannot stabilize the CyINPs at a certain total amount of polymer, which leads to the overall CyINPs particle size becoming larger. Meanwhile, the zeta potential decreases with increasing Fmoc-L-Phe ratio because Fmoc-L-Phe ions carry negative charges, causing CyINPs to display a more negative overall potential (Figure S14). As shown in Figure 1c,d, the absorbance of CyINPs increases and the fluorescence intensity decreases, while the concentration of Fmoc-L-Phe increases. This is due to the strong ACQ effect of CyI. Since the particle sizes of CyINPs are all around 90 nm–110 nm, and the corresponding encapsulation efficiency varies relatively large from 10% to around 50%, the more CyI dye is loaded, the more aggregated the CyI is, which leads to the weaker fluorescence intensity of CyINPs. In addition, the absorbance of Fmoc-L-Phe CyINPs is significantly higher than that of L-Phe CyINPs, which is attributed to the stronger interaction force between Fmoc-L-Phe and CyI, resulting in a higher absorbance of CyINPs. Although the encapsulation efficiency of CyI is increased by the increasing ratio of Fmoc-L-Phe, the fluorescence intensity of CyINPs is reduced subsequently. Therefore, in order to obtain a higher fluorescence intensity, the case of a 1:1 molar ratio is subsequently selected as the preparation condition.

### 3.3. Screening of CyI Concentrations

**Figure 2:**
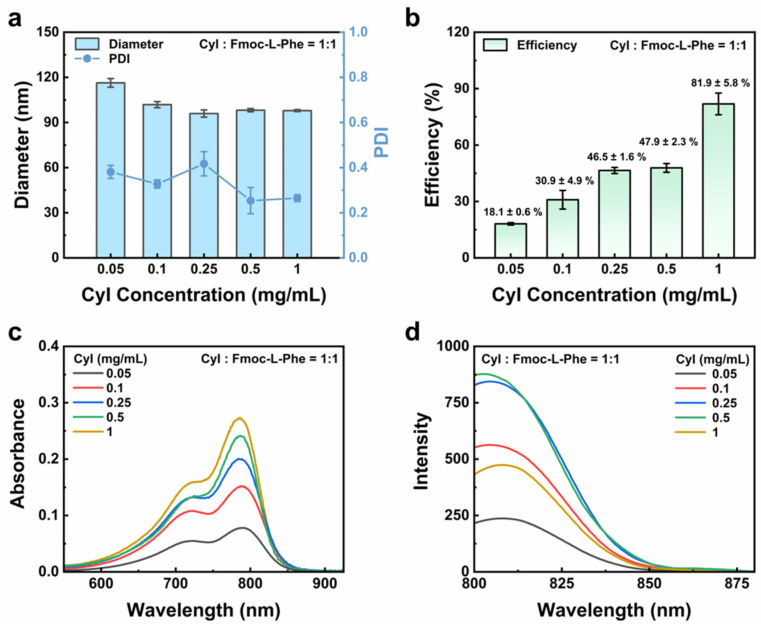
(**a**) Particle size, PDI, and (**b**) encapsulation efficiency of CyINPs with different CyI concentrations. (**c**) Absorption spectra and (**d**) emission spectra of CyINPs with different CyI concentrations ($\lambda_{ex}$ = 785 nm).

Under the condition of a fixed 1:1 molar ratio of CyI to counterions, different concentrations of CyI are varied to obtain higher encapsulation efficiency as well as stronger fluorescence signals. As shown in Figure 2a,b and Figure S15, the increase in CyI concentration leads to a gradual decrease in the particle size, accompanied by an increase in encapsulation efficiency. This can be attributed to the elevated supersaturation condition during the mixing process, resulting from the increase in the concentration of CyI, which makes the nucleation rate of the CyINPs surpass the growth rate, resulting in smaller CyINPs. As shown in Figure S16, the zeta potential gradually tends toward neutrality as the CyI concentration decreases. Additionally, the UV absorption intensity also becomes stronger with rising CyI concentration (Figure 2c). However, the intensity of the fluorescence signal exhibits a trend of initially increasing, followed by a subsequent decline (Figure 2d). At lower concentrations, the CyI dyes in the nanoparticles are in a relatively dispersed state and gradually aggregate as the concentration rises, without initially reaching saturation. As the concentration of CyI becomes large again, the CyI gradually aggregates, and the counterion is not enough to disperse the dye, thus making the fluorescence intensity weaker. The maximum fluorescence signal is observed at a CyI concentration of approximately 0.5 mg/mL, which also corresponds to a higher encapsulation efficiency. Accordingly, a concentration of 0.5 mg/mL is selected as the optimal CyI concentration for subsequent experiments.

### 3.4. Impact of Polymer Concentration on CyI Dispersion

**Figure 3:**
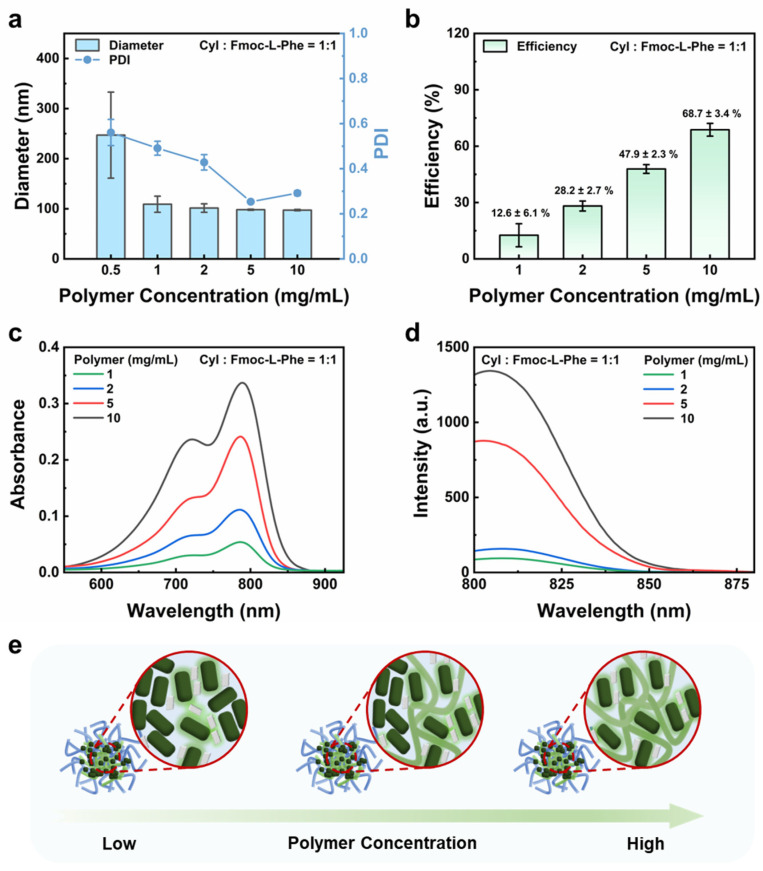
(**a**) Particle size, PDI, and (**b**) encapsulation efficiency of CyINPs with different polymer concentrations. (**c**) Absorption spectra and (**d**) emission spectra of CyINPs with different polymer concentrations ($\lambda_{ex}$ = 785 nm). (**e**) Schematic structure of CyINPs at different polymer concentrations.

By varying the polymer concentration from 0.5 mg/mL to 10 mg/mL while keeping the parameters constant, it can be observed that the increase in polymer concentration leads to a decrease in particle size of CyINPs, while concurrently the PDI of CyINPs also decreases, indicating improved CyINPs dispersion (Figure 3a and Figure S17). Since the PDI exceeds 0.6 at a polymer concentration of 0.5 mg/mL and visible sedimentation can be observed, this condition is not taken into account in the subsequent comparison of the encapsulation efficiency, absorbance, and fluorescence signals. The instability of CyINPs at low polymer concentration is attributed to the insufficient availability of polymer to effectively stabilize the hydrophobic core, thus making it difficult to form stable CyINPs. Whereas higher polymer concentrations provide an adequate amount of polymer to stabilize the nanoparticles, CyINPs formed with small particle sizes are more uniformly dispersed. As the amount of polymer increased, the zeta potential also became more negative (Figure S18). As shown in Figure 3b, as the polymer concentration increases, a greater number of CyI as well as counterion molecules are encapsulated, resulting in an enhancement in encapsulation efficiency of CyI. Correspondingly, it also leads to the enhancement of the absorbance of CyINPs with the increase in polymer concentration (Figure 3c). Notably, the fluorescence signal exhibits a more dramatic increase as the rising of polymer concentration rises (Figure 3d). This enhancement is due to the fact that the hydrophobic block of the polymer co-precipitates with CyI at higher polymer concentrations. This interaction between polymers and CyI dyes, along with the presence of counterions, facilitates the separation of CyI molecules, promoting a more dispersed state of CyI dye and thereby enhancing a stronger fluorescence signal.

### 3.5. Particle Performance of Optimized CyINPs

Fmoc-L-Phe is selected as the counterion, and the optimal molar ratio of CyI to Fmoc-L-Phe is established. Additionally, the concentrations of CyI and polymer were determined to be 0.5 mg/mL and 10 mg/mL, respectively. Under the optimized conditions, CyINPs are prepared by flash nanoprecipitation (FNP) and the thermodynamically controlled method (TM), respectively.

**Figure 4:**
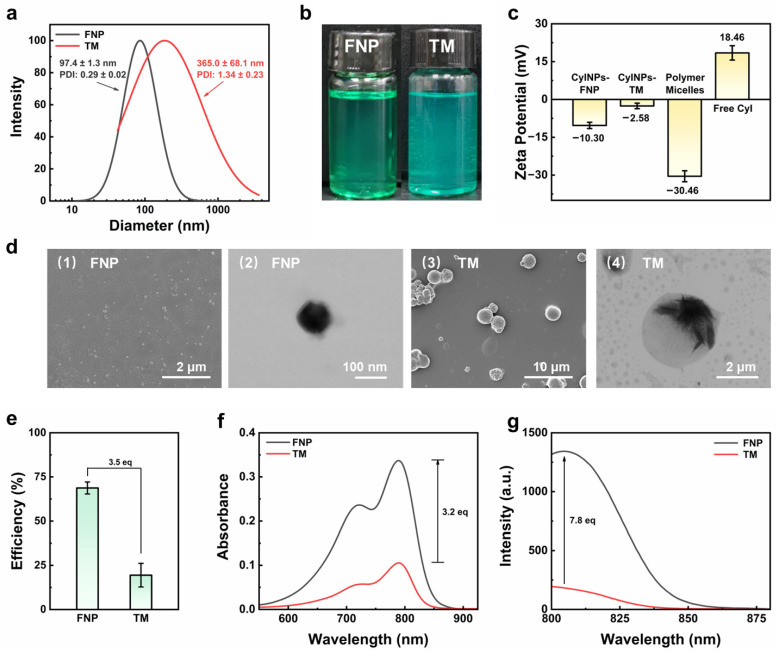
Photophysical properties of CyINPs. (**a**) Particle size distribution, (**b**) images, and (**c**) zeta potential of CyINPs-FNP and CyINPs-TM, respectively. (**d**) SEM and TEM images of CyINPs-FNP and CyINPs-TM. (**e**) Encapsulation efficiency of CyINPs-FNP and CyINPs-TM. (**f**) Absorption spectra and (**g**) emission spectra of CyINPs-FNP and CyINPs-TM ($\lambda_{ex}$ = 785 nm). Data are presented as mean ± standard deviation.

CyINPs prepared by the FNP method (CyINPs-FNP) exhibit an average particle size of approximately 98 nm with a PDI of around 0.3, resulting a CyINPs suspension that shows a greener color, closely resembling the color of CyI in the dispersed state (Figure 4a,b and Figure S19). In contrast, the particle size of CyINPs prepared by the TM (CyINPs-TM) is more than 350 nm with a PDI more than 1. Moreover, visible sedimentation is observed in CyINPs-TM. As shown in Figure 4c, the absolute value of CyINPs-FNP is greater than that of CyINPs-TM, resulting in more stable NPs. Meanwhile, it was also observed that amorphous CyINPs-FNP are formulated through the FNP method since the characteristic crystalline peaks of CyI and Fmoc-L-Phe disappeared in the CyINPs-FNP trace (Figure S20). TGA results (Figure S21) show that CyINPs-FNP have a much higher polymer weight content as compared with CyINPs-TM, which provide better protection of the CyI from water access, resulting in more stable NPs. As shown in Figure 4d and Figure S22, SEM and TEM images further confirm that CyINPs-FNP exhibit smaller and more uniformly dispersed nanoparticles, whereas CyINPs-TM display aggregated particles with a size of more than 2 μm. Additionally, a substantial number of smaller particles around 500 nm are observed around these larger aggregates, indicating poor dispersion of CyINPs-TM. As shown in Figure S23, particle sizes remain stable in urea solutions, demonstrating CyINPs formation is not caused by hydrogen bonding interactions. In contrast, particle sizes increase in NaCl, indicating disruption of electrostatic interactions, and decrease with Triton X-100, suggesting hydrophobic interaction in the CyINPs preparation. Therefore, electrostatic and hydrophobic interactions play important roles in CyINPs formation.

Furthermore, CyINPs-FNP exhibit significantly higher encapsulation efficiency and absorbance, both approximately 3.5-fold greater than CyINPs-TM (Figure 4e,f). After normalization, the fluorescence intensity of CyINPs-FNP is enhanced 7.8-fold compared with CyINPs-TM (Figure 4g). Additionally, the fluorescence quantum yield of CyINPs-FNP is 2-fold higher than CyINPs-TM (Table S1). This is because the TM method is thermodynamically driven, and CyI especially tends to aggregate easily; thus, CyI dyes obtained by TM are more likely to aggregate in the nanoparticles, leading to weaker fluorescence signals. On the other hand, the FNP method is kinetically driven, and CyINPs utilize the hydrophobic blocking of amphiphilic polymers as well as the co-precipitation and complexation of counterions to separate the CyI, obtaining higher encapsulation efficiency and stronger fluorescence signals.

### 3.6. Cell Cytotoxicity and Photothermal Effect of CyINPs-FNP

**Figure 5:**
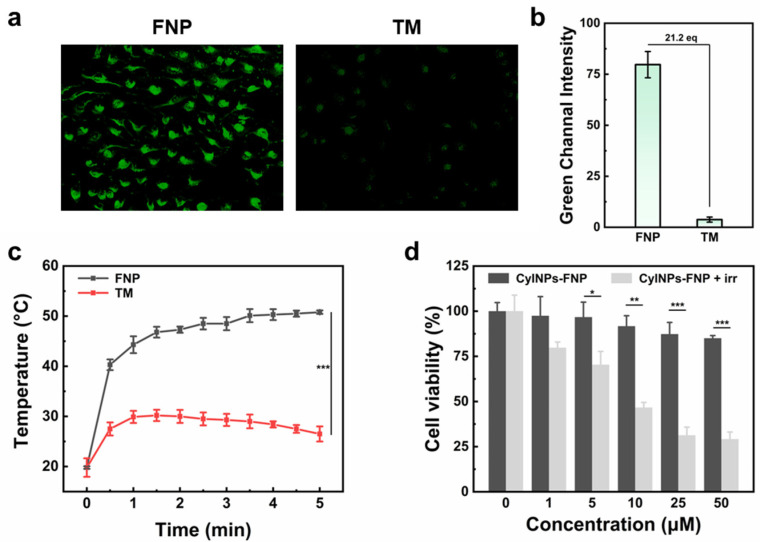
In vitro phototherapy performance of CyINPs-FNP. (**a**) CLSM images and (**b**) corresponding green channel intensity of fluorescence for HeLa cells upon CyINPs-FNP and CyINPs-TM treatments. (Scale bar: 20 μm) (**c**) Photothermic heating curves of CyINPs-FNP and CyINPs-TM under 808 nm irradiation (0.8 W∙cm^−2^) for 5 min. (**d**) HeLa cell viability after treatment with different concentrations of CyINPs-FNP with and without 808 nm irradiation (0.8 W∙cm^−2^). Data are presented as mean ± standard deviation. *p*-values are calculated by using one-way ANOVA with Tukey’s test, * *p* < 0.05, ** *p* < 0.01, *** *p* < 0.001.

Following the characterization of CyINPs’ photophysical properties, the cellular uptake and cytotoxicity of CyINPs are investigated. CyINPs-FNP and CyINPs-TM are incubated with HeLa cells for 12 h, after which intracellular fluorescence signals of HeLa cells are evaluated by confocal laser scanning microscopy (CLSM). As shown in Figure 5a,b, CyINPs-FNP exhibit greater cellular uptake, with fluorescence signals in the green channel enhanced more than 21-fold compared to CyINPs-TM. This is due to the fluorescence signal of CyINPs-FNP itself being greater than that of CyINPs-TM and the more efficient cellular uptake of CyINPs-FNP.

The introduction of the Fmoc group, which engages in the π-π interaction with CyI, imparts photothermal properties to CyINPs-FNP. In contrast to L-Phe as counterions of CyINPs that nearly show photothermal activity (Figure S24), the presence of the Fmoc group enables a significant photothermal effect of CyINPs-FNP, with the temperature rising to approximately 50 °C upon laser irradiation—suitable for photothermal therapy applications (Figure 5c). After 5 min of 808 nm laser irradiation, cell viability decreased to below 30%, indicating effective photothermal ability, while the survival rate of HeLa cells without laser irradiation remained above 85%, demonstrating good biocompatibility of the CyINPs-FNP (Figure 5d and Figure S25).

## 4. Conclusions

In this work, CyI fluorescent nanoparticles (CyINPs) are prepared by the FNP method with the introduction of block copolymers and counterions, realizing the loading of extremely hydrophobic ACQ cyanine dyes, which successfully improves the fluorescence intensity and encapsulation efficiency of CyINPs. The effects of different counterion concentrations on the stability and the enhancement of the fluorescence signal of CyINPs are investigated. There is an optimized process of CyINPs preparation, which involves more polymers and the proper counterion ratio, leading to the more dispersed CyI dyes in CyINPs, causing stronger CyINPs fluorescence intensity. Additionally, compared to the thermodynamic-controlled method, CyINPs prepared by the FNP method achieve a 21-fold higher fluorescence intensity and more efficient photothermal therapy, which provides ideas for improving the biocompatibility of the fluorescent nanoparticles and the efficiency of the utilization of energy.

## Data Availability

The original contributions presented in this study are included in the article/Supplementary Materials. Further inquiries can be directed to the corresponding authors.

## Supplementary Materials

The supporting information can be downloaded at: https://www.mdpi.com/article/10.3390/polym17152152/s1. Figure S1: The calibration curve of CyI; Figure S2: (**a**) Structure of CyI dye. (**b**) Free CyI dyes in different solvents including H_2_O, THF, and DMSO. (**c**) Absorption spectra of free CyI with different DMSO/H2O ratios. (**d**) Emission spectra of free CyI with different DMSO/H_2_O ratios ($\lambda_{ex}$ = 785 nm); Figure S3: (**a**) Size distribution, (**b**) particle size, PDI, and (**c**) images of CyINPs with different counterions before dialysis; Figure S4: (**a**) Images, (**b**) size distribution, (**c**) particle size, PDI, and (**d**) particle stability of CyINPs with different counterions after dialysis; Figure S5: Encapsulation efficiency of BARF-CyINPs and TPB-CyINPs after dialysis; Figure S6: (**a**) Absorption spectra and (**b**) emission spectra of BARF-CyINPs, TPB-CyINPs, and free CyI dye ($\lambda_{ex}$ = 785 nm); Figure S7: (**a**) Particle size, PDI, (**b**) size distribution, and (**c**) images of TPB-CyINPs with different CyI–TPB mole ratios; Figure S8: Encapsulation efficiency of TPB-CyINPs with different CyI–TPB mole ratios; Figure S9: (**a**) Absorption spectra and (**b**) emission spectra of TPB-CyINPs with different CyI–TPB mole ratios ($\lambda_{ex}$ = 785 nm); Figure S10: (**a**) Particle size, PDI, (**b**) size distribution, and (**c**) images of L-Phe CyINPs with different CyI–L-Phe mole ratios; Figure S11: Encapsulation efficiency of L-Phe CyINPs with different CyI–L-Phe mole ratio; Figure S12: (**a**) Absorption spectra and (**b**) emission spectra of L-Phe CyINPs with different CyI–L-Phe mole ratios ($\lambda_{ex}$ = 785 nm); Figure S13: (**a**) Size distribution and (**b**) images of Fmoc-L-Phe CyINPs with different CyI–Fmoc-L-Phe mole ratios; Figure S14: Zeta potential of CyINPs with different CyI–Fmoc-L-Phe mole ratios; Figure S15: Size distribution of Fmoc-L-Phe CyINPs with different CyI concentrations; Figure S16: Zeta potential of CyINPs with different CyI concentrations; Figure S17: (**a**) Size distribution and (**b**) images of Fmoc-L-Phe CyINPs with different polymer concentrations; Figure S18: Zeta potential of CyINPs with different polymer concentrations; Figure S19: Stability of particle size of CyINPs-FNP and CyINPs-TM, respectively; Figure S20: XRD of CyINPs-FNP, Fmoc-L-Phe, and CyI, respectively; Figure S21: TGA of CyINPs-FNP and CyINPs-TM, respectively; Figure S22: TEM images of (**a**,**b**) polymer micelles, (**c**,**d**) CyINPs-FNP, and (**e**,**f**) CyINPs-TM; Figure S23: Size and PDI variations of CyINPs after dispersion in NaCl, urea, and Triton X-100 solutions; Figure S24: Photothermic heating curves of L-Phe CyINPs under 808 nm irradiation (0.8 W∙cm^−2^) for 5 min; Figure S25: HeLa cell viability after treatment with different concentrations of CyINPs-TM with and without 808 nm irradiation (0.8 W∙cm^−2^); Figure S26: ^1^H-NMR spectrum of CyI in CDCl_3_; Table S1: Fluorescence quantum yield of CyINPs-FNP.
