# Supplementary material for: Controllable Structure and Fluorescence Enhancement of ACQ Dye Nanoparticles Based on the FNP Process
Source: Polymers (Basel). 2025 Aug 6;17(15):2152. doi: 10.3390/polym17152152 (PMC12349522; doi:10.3390/polym17152152)
Supplement: Supplementary file 1 [file polymers-17-02152-s001.zip › polymers-3719834-supplementary.pdf]

Supporting information

Controllable structure and fluorescence enhancement of ACQ dye nanoparticles based on the FNP process

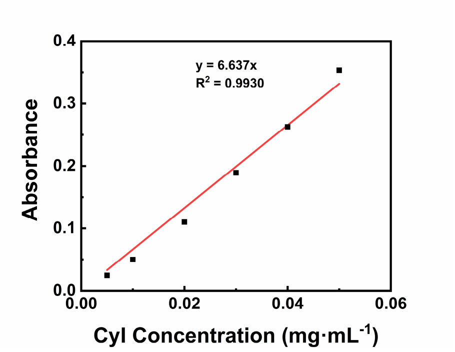

Figure S1. The calibration curve of CyI.

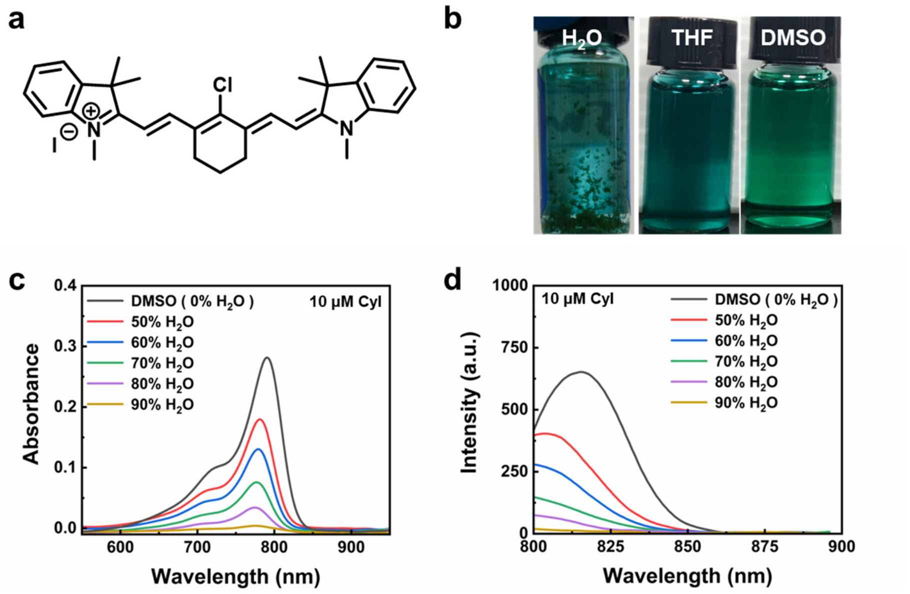

Figure S2. (**a**) Structure of CyI dye. (**b**) Free CyI dyes in different solvents including H_2O, THF, and DMSO. (**c**) Absorption spectra of free CyI with different DMSO/H2O ratios. (**d**) Emission spectra of free CyI with different DMSO/H_2O ratios (λ_{ex}= 785 nm).

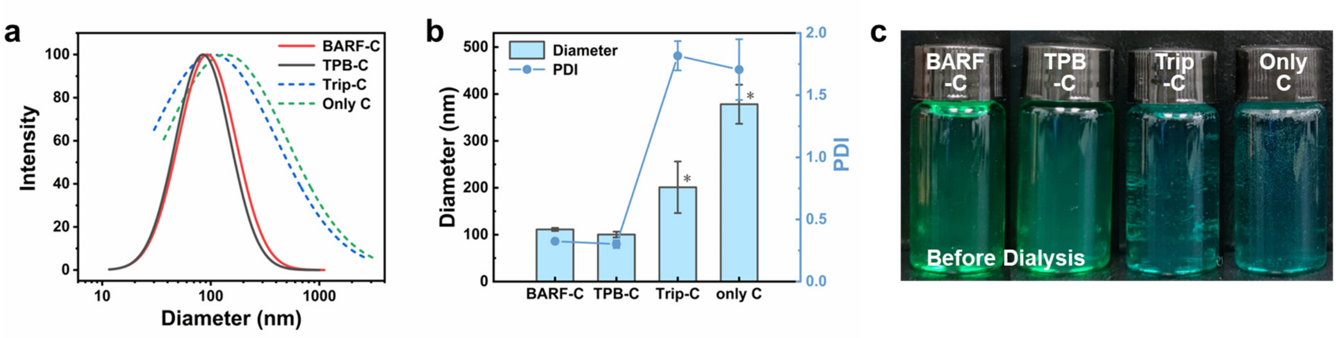

Figure S3. (**a**) Size distribution, (**b**) particle size, PDI, and (**c**) images of CyINPs with different counterions before dialysis.

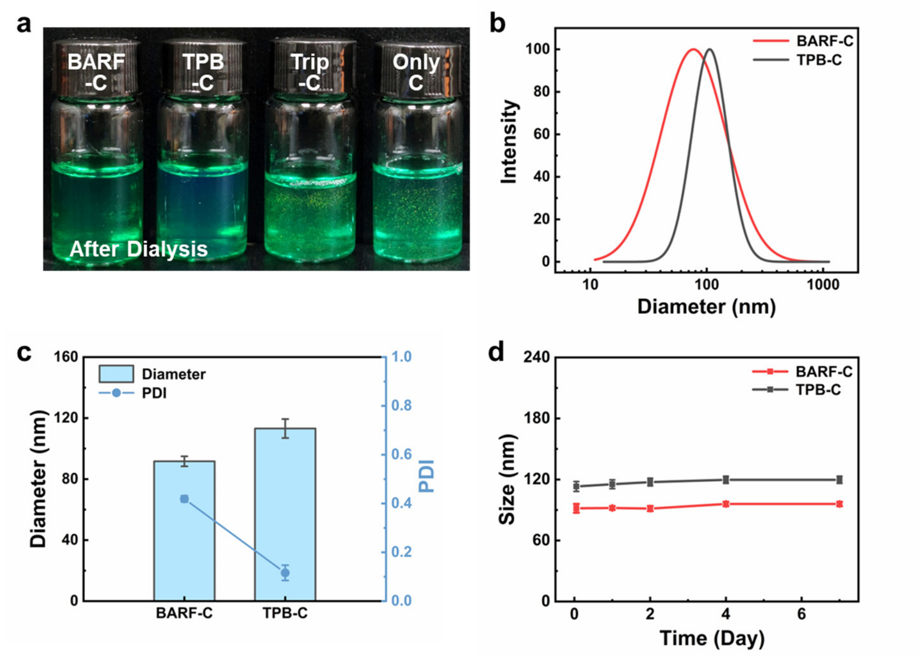

Figure S4. (**a**) Images, (**b**) size distribution, (**c**) particle size, PDI, and (**d**) particle stability of CyINPs with different counterions after dialysis.

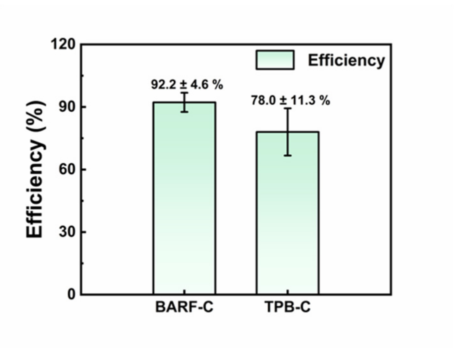

Figure S5. Encapsulation efficiency of BARF-CyINPs and TPB-CyINPs after dialysis.

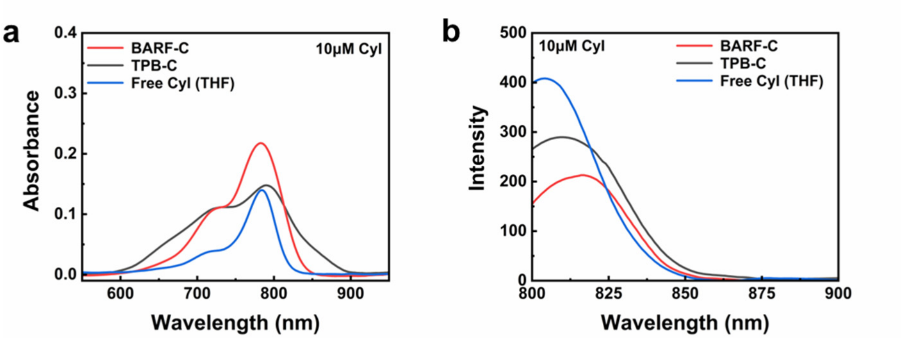

Figure S6. (**a**) Absorption spectra and (**b**) emission spectra of BARF-CyINPs, TPB-CyINPs, and free CyI dye (λ_{ex}= 785 nm).

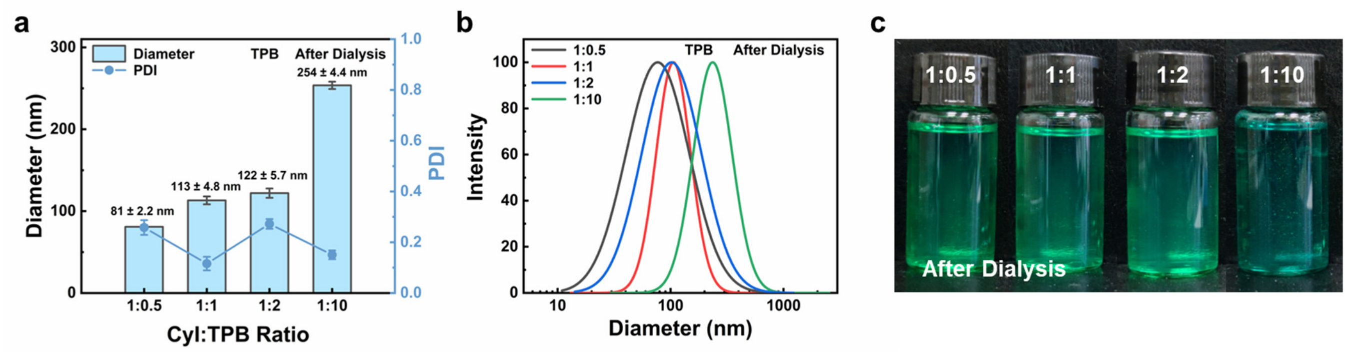

Figure S7. (**a**) Particle size, PDI, (**b**) size distribution, and (**c**) images of TPB-CyINPs with different CyI–TPB mole ratios.

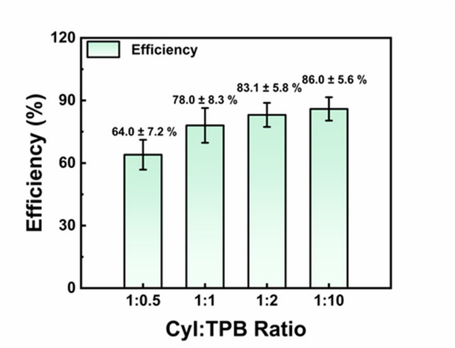

Figure S8. Encapsulation efficiency of TPB-CyINPs with different CyI–TPB mole ratios.

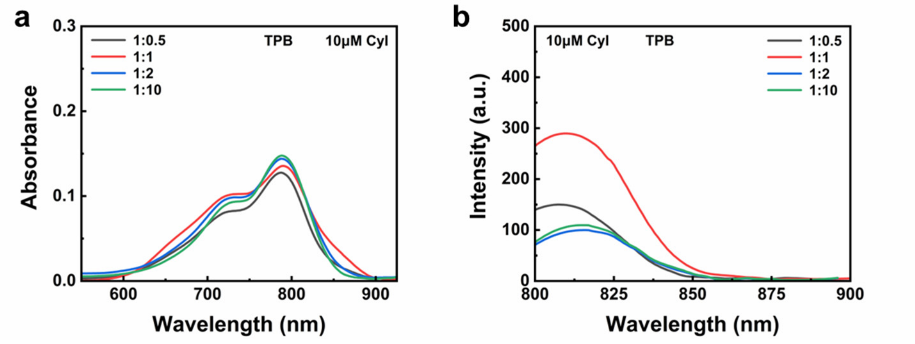

Figure S9. (**a**) Absorption spectra and (**b**) emission spectra of TPB-CyINPs with different CyI–TPB mole ratios (λ_{ex}= 785 nm).

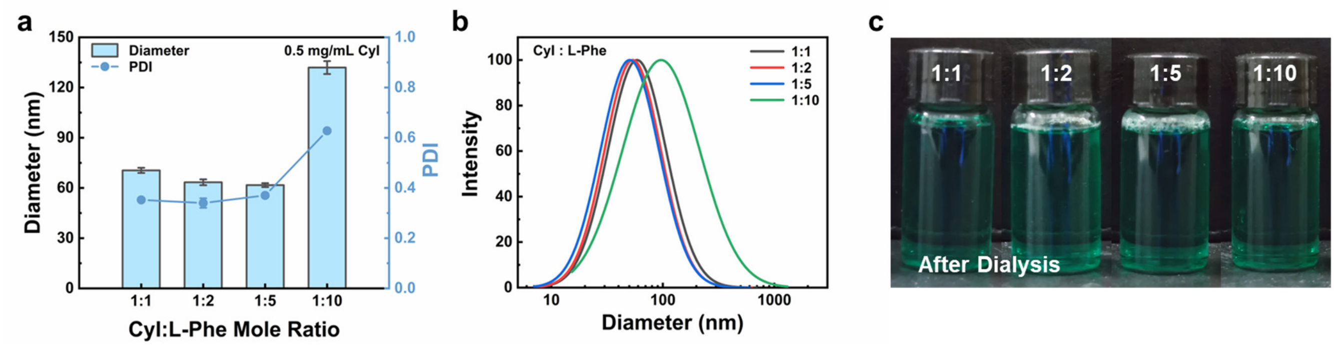

Figure S10. (**a**) Particle size, PDI, (**b**) size distribution, and (**c**) images of L-Phe CyINPs with different CyI–L-Phe mole ratios.

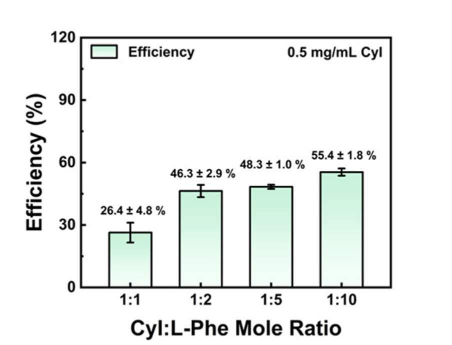

Figure S11. Encapsulation efficiency of L-Phe CyINPs with different CyI–L-Phe mole ratios.

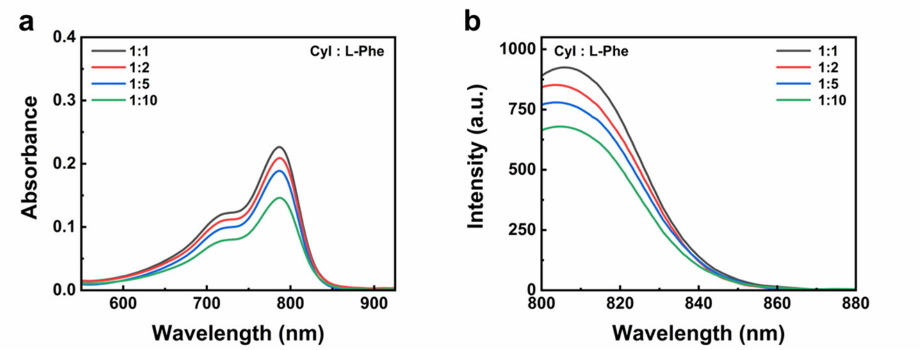

Figure S12. (**a**) Absorption spectra and (**b**) emission spectra of L-Phe CyINPs with different CyI–L-Phe mole ratios (λ_{ex}= 785 nm).

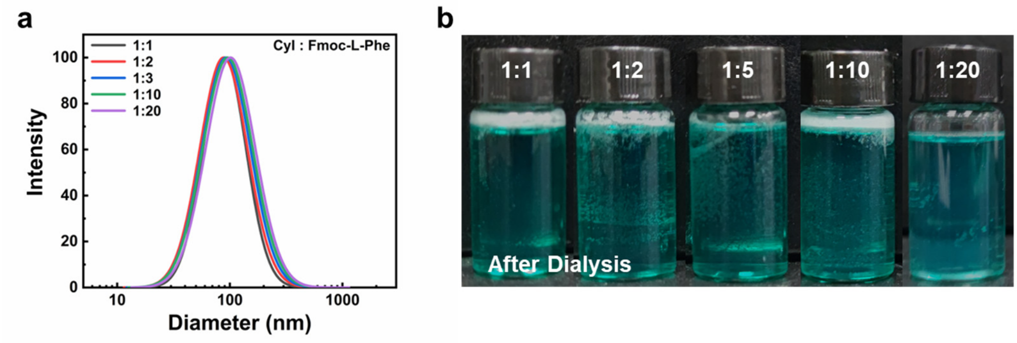

Figure S13. (**a**) Size distribution and (**b**) images of Fmoc-L-Phe CyINPs with different CyI–Fmoc-L-Phe mole ratios.

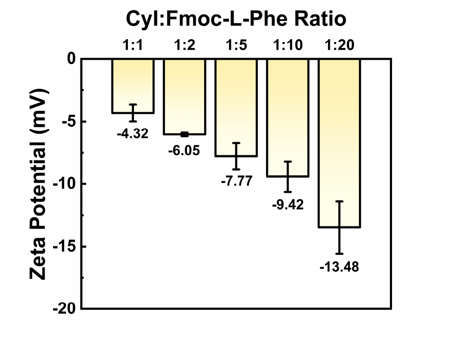

Figure S14. Zeta potential of CyINPs with different CyI–Fmoc-L-Phe mole ratios.

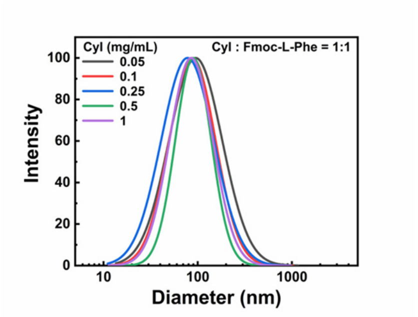

Figure S15. Size distribution of Fmoc-L-Phe CyINPs with different CyI concentrations.

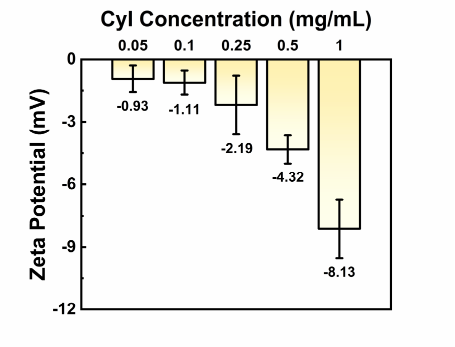

Figure S16. Zeta potential of CyINPs with different CyI concentrations.

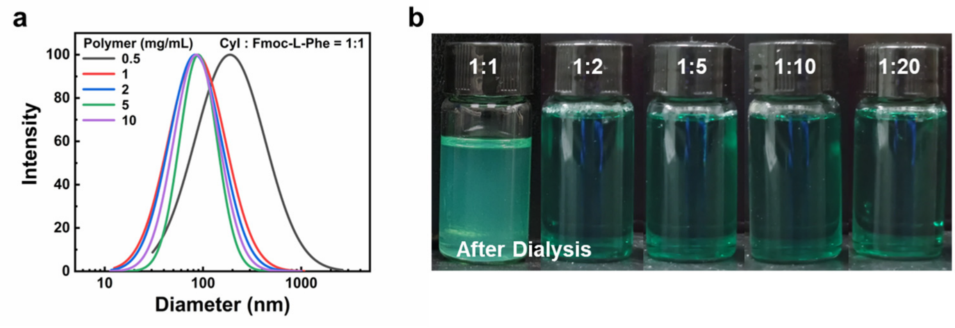

Figure S17. (**a**) Size distribution and (**b**) images of Fmoc-L-Phe CyINPs with different polymer concentrations.

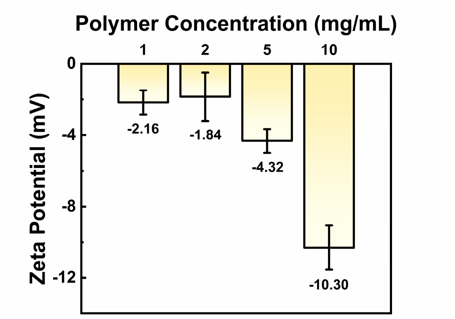

Figure S18. Zeta potential of CyINPs with different polymer concentrations.

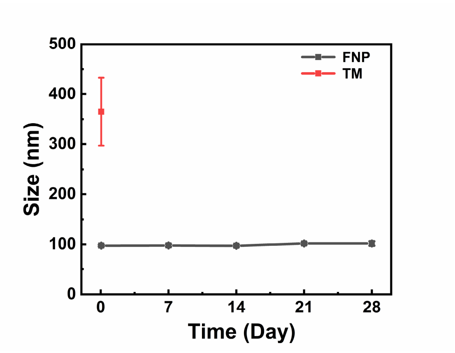

Figure S19. Stability of particle size of CyINPs-FNP and CyINPs-TM, respectively.

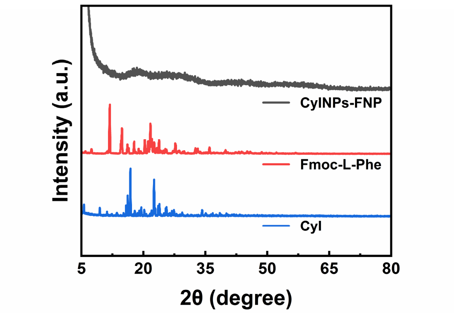

Figure S20. XRD of CyINPs-FNP, Fmoc-L-Phe, and CyI, respectively.

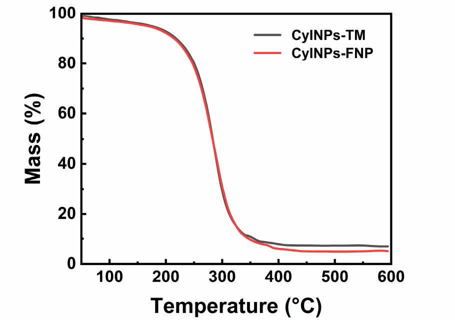

Figure S21. TGA of CyINPs-FNP and CyINPs-TM, respectively.

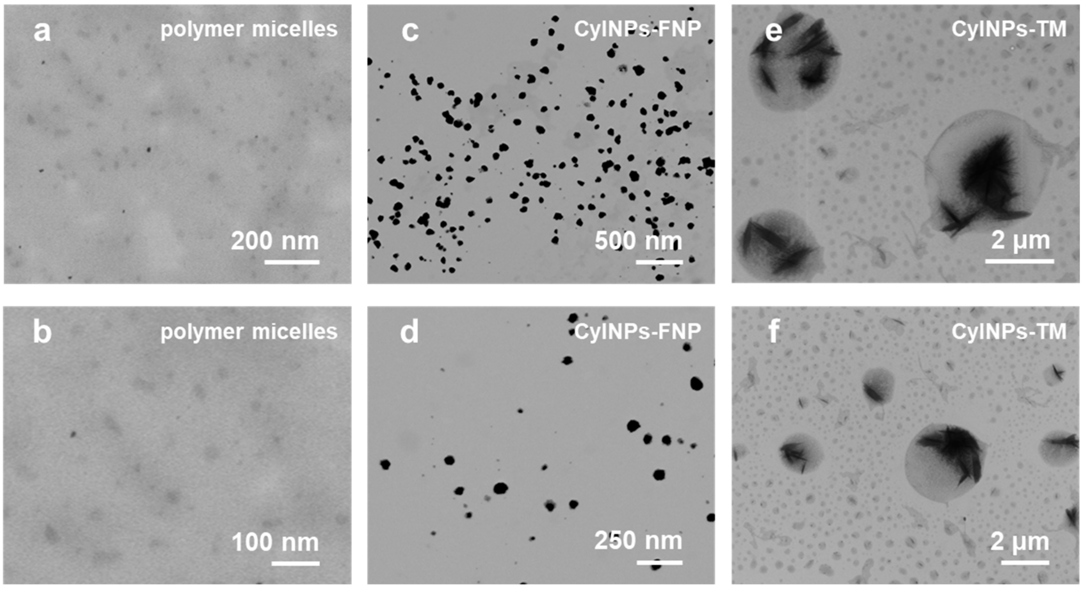

Figure S22. TEM images of (**a**,**b**) polymer micelles, (**c**,**d**) CyINPs-FNP, and (**e**,**f**) CyINPs-TM.

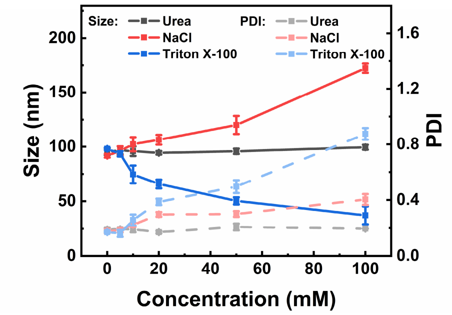

Figure S23. Size and PDI variations of CyINPs after dispersion in NaCl, urea, and Triton X-100 solutions.

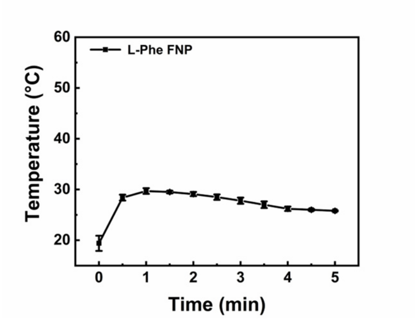

Figure S24. Photothermic heating curves of L-Phe CyINPs under 808 nm irradiation (0.8 W·cm^{-2}) for 5 min.

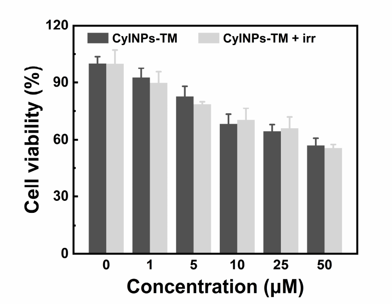

Figure S25. HeLa cell viability after treatment with different concentrations of CyINPs-TM with and without 808 nm irradiation (0.8 W·cm^{-2}).

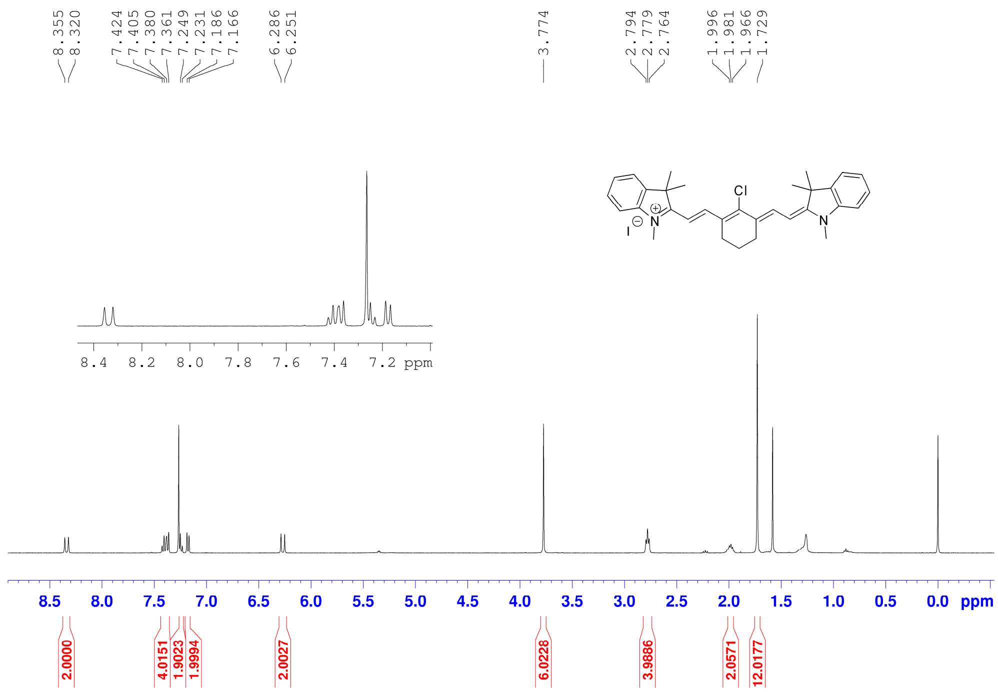

Figure S26. ^{1}H-NMR spectrum of CyI in $CDCl_3$.

Table S1. Fluorescence quantum yield of CyINPs-FNP.

	CyINPs-FNP	CyINPs-TM	CyI	ICG
Φ	20.7	10.9	19.3	12.8
